# Priority research directions for wildfire science: views from a historically fire-prone and an emerging fire-prone country

**DOI:** 10.1098/rstb.2024.0001

**Published:** 2025-04-17

**Authors:** Kerryn Little, Rayanne Vitali, Claire M. Belcher, Nicholas Kettridge, Adam F.A. Pellegrini, Adriana E.S. Ford, Alistair M.S. Smith, Andy Elliott, Apostolos Voulgarakis, Cathelijne R. Stoof, Crystal A. Kolden, Dylan W. Schwilk, Eric B. Kennedy, Fiona E. Newman Thacker, Gail R. Millin-Chalabi, Gareth D. Clay, James I. Morison, Jessica L. McCarty, Katy Ivison, Kevin Tansey, Kimberley J. Simpson, Matthew W. Jones, Michelle C. Mack, Peter Z. Fulé, Rob Gazzard, Sandy P. Harrison, Stacey New, Susan E. Page, Tilly E. Hall, Tim Brown, W. Matt Jolly, Stefan Doerr

**Affiliations:** ^1^ School of Geography, Earth and Environmental Sciences, University of Birmingham, Birmingham, UK; ^2^ WildFIRE Lab, University of Exeter, Exeter, Devon, UK; ^3^ iClimate, Department of Environmental Science, Aarhus University, Aarhus, Midtjylland, Denmark; ^4^ University of Cambridge, Cambridge, UK; ^5^ Leverhulme Centre for Wildfires, Environment and Society, Imperial College London, London, UK; ^6^ Department of Earth and Spatial Sciences, University of Idaho, Moscow, ID, USA; ^7^ School of Chemical and Environmental Engineering, Technical University of Crete, Chania, Greece; ^8^ Soil Physics and Land Management Group, Wageningen University & Research, Wageningen, The Netherlands; ^9^ University of California Merced School of Engineering, Merced, CA, USA; ^10^ Texas Tech University, Lubbock, TX, USA; ^11^ School of Administrative Studies, York University, North York, Canada; ^12^ Soil Physics and Land Management Group, Wageningen University and Research, Wageningen, The Netherlands; ^13^ Department of Geography, University of Manchester, Manchester, UK; ^14^ Forest Research UK, Farnham, UK; ^15^ NASA Ames Research Center, Moffett Field, CA, USA; ^16^ School of Geography, Geology and the Environment, University of Leicester, Leicester, UK; ^17^ Animal and Plant Sciences, University of Sheffield, Sheffield, UK; ^18^ Botany Department, Rhodes University, Grahamstown, South Africa; ^19^ School of Environmental Sciences, University of East Anglia, Norwich, UK; ^20^ Center for Ecosystem Science and Society, Department of Biological Sciences, Northern Arizona University, Flagstaff, AZ, USA; ^21^ School of Forestry, Northern Arizona University, Flagstaff, AZ, USA; ^22^ Forestry Commission England, Brandon, UK; ^23^ Defra, London, UK; ^24^ Geography and Environmental Science, University of Reading, Reading, UK; ^25^ Met Office Hadley Centre, Exeter, UK; ^26^ Department of Geography, Durham University, Durham, UK; ^27^ Desert Research Institute, Reno, Nevada, UK; ^28^ Rocky Mountain Research Station Missoula Fire Sciences Laboratory, Missoula, MT, USA; ^29^ Department of Geography, Swansea University, Swansea, UK

**Keywords:** wildfire, fire behaviour, research gaps, fire ecology, fire regimes, global change

## Abstract

Fire regimes are changing across the globe, with new wildfire behaviour phenomena and increasing impacts felt, especially in ecosystems without clear adaptations to wildfire. These trends pose significant challenges to the scientific community in understanding and communicating these changes and their implications, particularly where we lack underlying scientific evidence to inform decision-making. Here, we present a perspective on priority directions for wildfire science research—through the lens of academic and government wildfire scientists from a historically wildfire-prone (USA) and emerging wildfire-prone (UK) country. Key topic areas outlined during a series of workshops in 2023 were as follows: (A) understanding and predicting fire occurrence, fire behaviour and fire impacts; (B) increasing human and ecosystem resilience to fire; and (C) understanding the atmospheric and climate impacts of fire. Participants agreed on focused research questions that were seen as priority scientific research gaps. Fire behaviour was identified as a central connecting theme that would allow critical advances to be made across all topic areas. These findings provide one group of perspectives to feed into a more transdisciplinary outline of wildfire research priorities across the diversity of knowledge bases and perspectives that are critical in addressing wildfire research challenges under changing fire regimes.

This article is part of the theme issue ‘Novel fire regimes under climate changes and human influences: impacts, ecosystem responses and feedbacks’.

## 1. Introduction

Fire has been a natural disturbance in many ecosystems for millions of years [1]. Landscape fires (including uncontrolled wildfires and managed burns) burn over 7 million km^2^ globally each year (an area equivalent in size to Australia), with ignitions mostly by lightning or humans [2]. Climate change has lengthened the annual periods when weather conditions are conducive to wildfire by over 25% in the last four decades across nearly all regions of the world, with climate models predicting an accelerated increase in the coming decades [3,4]. Wildfires now affect ecosystems with species that have no clear evolutionary or societal adaptations to fire such as some tropical rainforests, deserts, riparian gallery forests and the high-latitude Arctic tundra [5]. This can lead to lasting ecosystem degradation and threaten to turn boreal and tropical forests, as well as peatland ecosystems, from net carbon sinks to sources [4,6]. These changes threaten global efforts to reach net zero and enhance anthropogenic impacts on emission inventories, such that wildfires currently emit approximately 2 Gt of carbon per year (equivalent to approximately one-fifth of anthropogenic emissions) [7]. Fire emissions from forest and peatland ecosystems have been increasing since 2000 [4]. The 2023 wildfires in Canada alone emitted almost 480 megatons of carbon and represented 23% of the global total carbon emissions for 2023 [8]. These emissions will not be absorbed by the regrowing vegetation for decades, or even centuries in the case of peatlands, leading to a positive carbon–climate warming–fire feedback [8].

The occurrence of ‘extreme wildfire’ events, which are particularly severe in terms of their size, duration, intensity and impacts, is also on the rise [9]. In the forests of the western USA, wildfire severity increased eightfold between 1985 and 2017 [10]. Extreme wildfires have significant impacts on human lives and well-being, on ecosystems, the climate system and the economy [11,12]. Despite advances in communication, firefighting and evacuation, the average direct annual human death toll due to wildfire has more than doubled since 2020 globally compared to the previous decades [13], and annual premature deaths from wildfire-derived air pollution are estimated to exceed 340 000 globally [14].

The economic impacts of extreme wildfires are rising to unprecedented levels both in countries with a long history of wildfire and those where wildfire is becoming an increasing disturbance. The 2019−2020 wildfires in Australia caused USD 23 billion in direct economic damages and the 2018 ‘Camp Fire’ in California alone cost USD 19 billion—the costliest in both countries’ histories [13,15]. In the UK, unprecedented fire weather conditions in June 2022 [16] resulted in the first significant ingress of wildfire into urban regions with the loss of over 70 structures in a series of relatively small crop and grassland wildfires in London [17]. While economic losses from wildfires globally remain lower than those from other major disturbances such as storms, floods or earthquakes, they have become the most expensive natural hazard per person affected [18]. The economic costs of the 2023 Lahaina wildfires in Hawaii that led to the highest USA wildfire death toll in a century are estimated to be up to USD 16 billion [19]. In California, destructive wildfires have led to several insurance companies not renewing or offering wildfire insurance policies in 2023 to households in affected areas, a move that forced the state government to consider relaxing rules regarding increased premiums for future climate change conditions [20,21].

Extreme wildfire activity and associated negative impacts are expected to rise further over the 21st century, alongside continued climate change, land management practices that often favour fuel accumulation, and population changes further increasing ignitions and human exposure to wildfire [3,18,22]. The rapid pace of changing wildfire activity globally is a significant challenge to the scientific community, in both understanding and communicating changing wildfire patterns, behaviours and their implications. Given the shifts in wildfire activity and its increasingly devastating impacts, the need to support innovative solutions, fund research and adopt policy to address wildfire-related challenges continues to grow [22].

The changes in wildfire activity and impact introduce new challenges for which we depend on a robust knowledge base to inform decision-making processes. As such, we need to redefine wildfire research agendas that span the diversity of perspectives and expertise across broad disciplines, geographies and cultures, especially valuing local, traditional and Indigenous knowledges [23–25]. One small facet of this broader wildfire research agenda is the need to increase international collaboration between academic and government science researchers across historically wildfire-prone and emerging wildfire-prone countries. Increased international collaboration can support the integration of extensive, advanced, long-term research approaches with expertise from new wildfire ecosystems or specialized centres of expertise, as well as associated data-sharing opportunities. In this opinion piece, we present perspectives on new directions for wildfire research, as viewed by the scientific research communities of exemplar, historically wildfire-prone and emerging wildfire-prone countries. We asked UK- and USA-based wildfire researchers across the disciplines of physical sciences and engineering what they see as the global wildfire research challenges through a series of workshops held in 2023. In doing so, we outline areas of research priorities and funding in wildfire science as viewed by this community, considering synergies where shared learning could address these global research gaps. These findings provide one perspective to feed into a more transdisciplinary outline of wildfire research priorities across the diversity of knowledge bases and perspectives that are needed to address the wildfire challenges of the future.

## 2. Methods

We held targeted workshops with UK- and USA-based wildfire science researchers within academic and government institutions. These workshops specifically targeted a small subset of the broader range of perspectives from communities and disciplines that conduct wildfire research to present a perspective on wildfire science research needs between an exemplar, historically wildfire-prone and emerging wildfire country where there are opportunities for synergistic advances. UK- and USA-based researchers were asked to consider global wildfire research challenges (not just in their home country or own field of expertise). We recognize that the workshop outcomes are represented through this lens, and it should be kept in mind that these perspectives do not represent the full diversity of wildfire expertise at an international and transdisciplinary scale.

**Figure 1.**
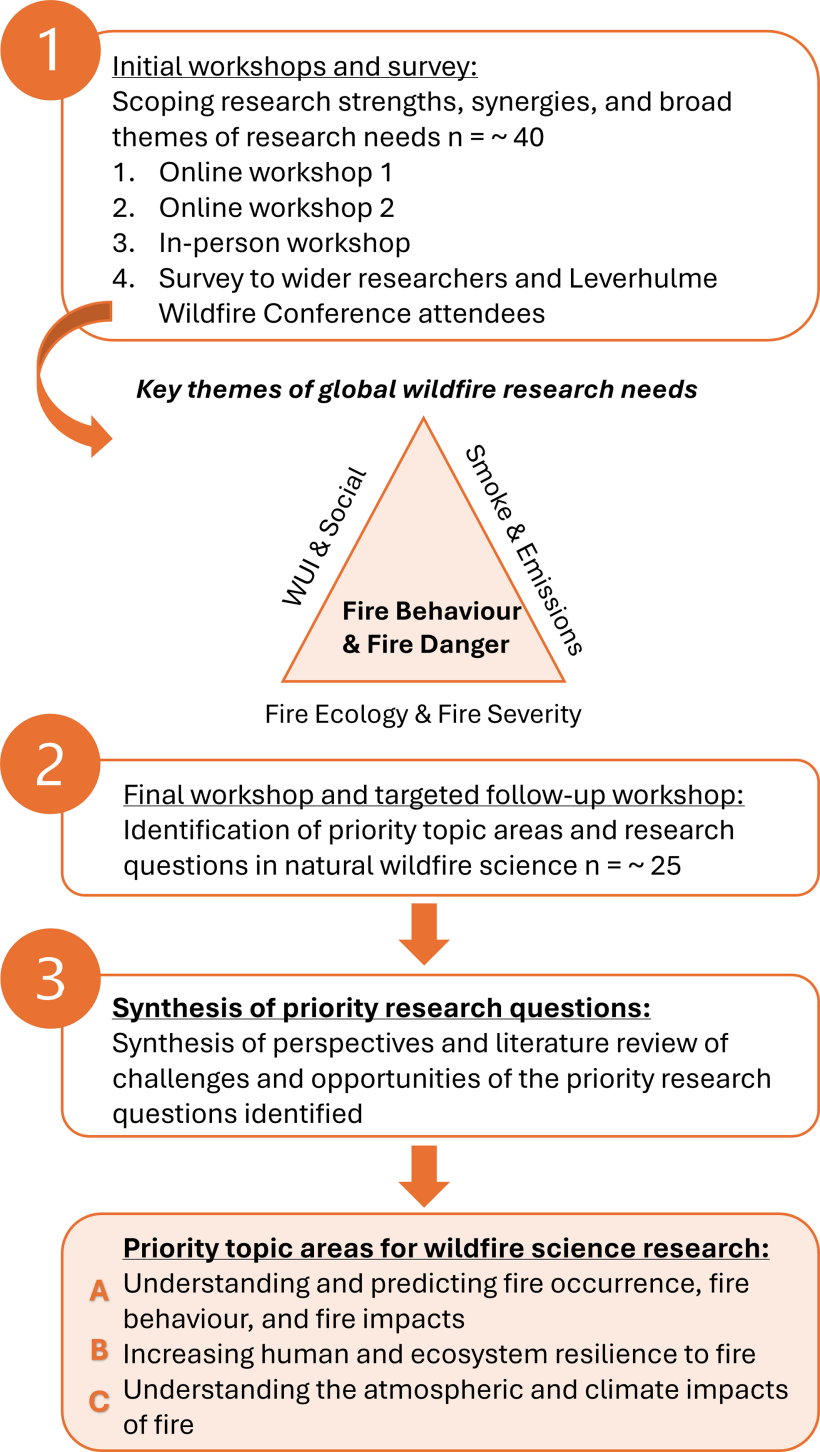
Methods employed to identify the priority research questions.

We invited participants to join one of three initial workshop events, attempting to capture the diversity of expertise held by UK- and USA-affiliated wildfire scientists working in academic and government research institutions (figure 1). We asked participants to highlight what they view to be key wildfire research challenges globally, by considering the two countries’ strengths, weaknesses and opportunities for synergistic research collaborations. During the workshops, we identified and agreed upon four broad themes: fire behaviour and fire danger, wildland–urban interface/rural–urban interface (WUI/RUI) and social themes, fire ecology and fire severity, and smoke and emissions. We then disseminated an online survey to a wider group of wildfire researchers and the attendants of the international Leverhulme Wildfire’s Summer Conference in July 2023, asking participants to outline key strengths that they feel already exist in wildfire research and one potential area where international collaboration could lead to transformative change.

We held a further workshop involving all of the participants collectively. As a group, we discussed the four broad themes and findings from the initial workshops and narrowed down three topic areas A–C (figure 1) that captured these themes and clearly identified perceived priority wildfire research needs (i.e. focused topic areas that discern the priority funding and research needs from the perspective of the workshop participants). Workshop participants also identified key research questions within the three priority topic areas that were perceived as key avenues for future research and funding in this sphere. A final focus group met to summarize these research priorities, and the research questions were used to guide a thematic literature review of research challenges and opportunities across the identified priority research questions.

## 3. Results

### (a) Overall perspectives on wildfire science research needs: results from initial general discussions

The participants of the workshops spanned a broad spectrum of wildfire science research fields. Despite their different specializations and geographic study regions and environments, there was agreement on the broad themes in wildfire science research requiring priority attention to address research gaps and challenges. The workshops comprised open discussions to encourage all ideas to be explored without limiting the direction of conversations. Discussions often centred around recent notable wildfire events and seasons that were perceived to highlight current research challenges.

Despite being primarily science-focused, all participants emphasized the need to improve our understanding of human–fire interactions (acknowledging that this has a diverse range of interpretations), with challenges such as minimizing structural damage, fire science communication and community-based fire and fuel management proposed as important areas of focus. The impacts of hugely destructive wildfires (e.g. Camp Fire, USA, 2018; Black Summer Fires, Australia, 2019−2020; Barrington Lake Fire, Canada, 2023), in addition to those in countries that have not seen large-scale damage before from wildfire (e.g. the destruction of homes in the Wennington Fire, UK, 2022) were at the forefront of discussions around these research challenges. The WUI/RUI is identified as a critical area of research globally, as it represents the nexus of human–fire interaction, where lives, homes and communities are often most vulnerable to wildfire [26]. Additionally, understanding human causes of ignitions, particularly arson, was highlighted as fundamental but not well understood. There was also a recognized need to improve our ability to model human–fire interactions within global models of fire regimes, incorporating people as agents of change beyond proxies like GDP and population density, to better inform fire-related policy and management [27,28].

Participants also emphasized how the extreme 2023 Canada wildfires (that occurred during the workshop period) exposed gaps in the scientific community’s understanding of smoke. The politics of smoke, smouldering fire smoke, air quality and atmospheric dispersion models for smoke were discussed as potential research gaps that have global implications. There was a consensus that current fire models are still relatively poor in respect to smoke prediction. There is an increasing need for their improvement in atmospheric dispersion models, fire behaviour models, models for fire spread through communities, and global fire models that are used as tools in wildfire research. Model improvement was viewed as being particularly important in respect to the extreme wildfire behaviour we are seeing in many countries globally, where smoke pollution has been a major issue (e.g. Western USA, 2023, Black Summer Australia, 2019−2020). Participants agreed that while many countries may work on improvements independently, transdisciplinary collaboration in this area would lead to more efficient progress.

Participants highlighted how, in many cases, research gaps exist between wildfire research on different scales. For example, models that are deemed sufficient on global scales were perceived by participants to be relatively poor at simulating on a regional scale, particularly in terms of fire behaviour and smoke dispersion. A common theme from workshop discussions was thus the need for a more universal view of fire as a global process, tying wildfire research on different spatial scales and across different disciplines together.

Workshop participants noted extensively that an improvement in fundamental knowledge of wildfire itself is required first to enable exploration of more complex questions, particularly due to climate- and human-driven changes in fire regimes. For example, model development and understanding of processes such as fire and fuel management, smoke and emissions largely rely on data that are either currently lacking or mostly outdated where it does exist, as also identified in [22]. Building an understanding of fire processes and the acquisition of useful data was therefore considered to be required as a base for all future wildfire research. Subjects identified as central to this foundation included improving our knowledge of changing fuel loads, fire behaviour, post-fire vegetation regrowth in different ecosystems, and refining definitions and associated measurements of fire severity. These foundations may enable expanding work on fire forecasting over different timescales and building improved fire danger rating systems for different regions, both in countries that have longstanding systems and those with none.

Another research challenge identified was the need to understand paleofire and deep time evolutionary traits, where better understanding of the history of fire is necessary to truly understand fire at present and in the future. Understanding plant traits that evolved in response to fire may help us predict if such species can persist in areas suffering extreme fire behaviour and increased fire frequency. Improving knowledge of fire ecology and behaviour was highlighted as key given the current global changes in which increased duration of fire weather, wildfire activity, and severity in global forests are already being observed [3,29]. Participants agreed that understanding how future change will alter ecosystems, fire regimes, fire behaviour and further impacts on the Earth system (e.g. on global feedbacks and biogeochemical cycles) is consequently essential for answering questions of how to adapt and mitigate against the changing wildfire regimes we face.

From the workshop discussions, workshop participants and organizers reflected on broad themes that captured the wildfire research challenges discussed. Participants agreed that discussions could be summarized into broad themes: (i) fire behaviour and fire danger; (ii) WUI/RUI; (iii) smoke and emissions; (iv) fire ecology and fire severity. It was noted during the workshops that many comments on research foci overlapped and fell into multiple themes, and almost all points touched on fire behaviour. Of key significance was the perspective that the behaviour of wildfires over the past 5+ years has been changing across the globe with extremes of fire behaviour never or rarely seen before now being observed in many regions (west coast of North America, Mediterranean, Australia). This includes very rapid fire spread (also at night-time) and more extreme spotting, crowning, deep flaming and pyroconvection. It was discussed how these ‘new fire phenomena’ could suggest we have passed a ‘climate–fire tipping point’ that our existing fire research base no longer suffices to describe. Changing fire behaviour means that both novel and renewed research in all key themes identified is required.

### (b) Perspectives on wildfire science topic areas and research question priorities identified via in-depth discussions

As a group, we discussed how the broad themes of fire behaviour and danger, WUI/RUI, smoke and emissions, and fire ecology and fire severity could be distilled into key topic areas that explicitly communicate where research is needed:

(A) understanding and predicting fire occurrence, fire behaviour and fire impacts;
(B) increasing human and ecosystem resilience to fire; and
(C) understanding the atmospheric and climate impacts of fire.

**Figure 2.**
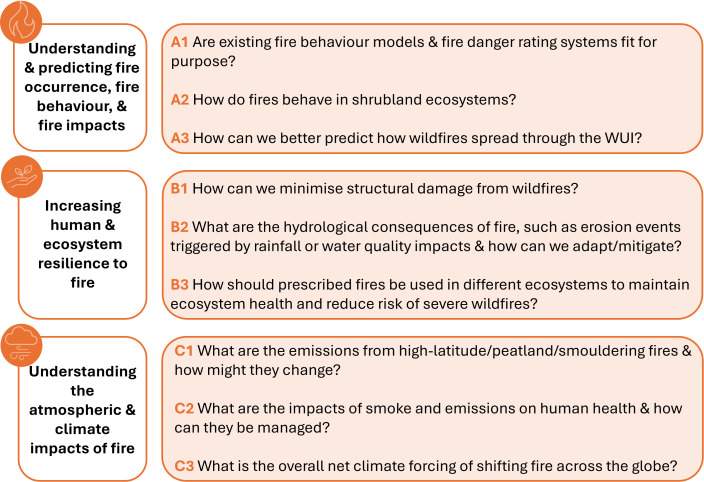
Overview of identified research questions and activities (right-hand side) within each topic area (left-hand side). Each topic area is relevant to all of the identified central themes: fire behaviour and fire danger, WUI/RUI, fire ecology and fire severity, and smoke and emissions.

Each topic area encompasses the broad wildfire science research themes discussed in the initial workshops, and in particular, participants discussed the role of fire behaviour as a central connecting theme across global processes. Within the perceived priority topic areas, participants identified specific research gaps/questions that should be targeted by funding bodies and wildfire scientists to address research challenges under changing fire regimes (figure 2). The following sections summarize these workshop outcomes in the context of the scientific literature.

#### (i) Predicting fire occurrence, behaviour and impacts

Fires are increasingly affecting areas where fires had been rare in the past or showing new extremes in fire behaviour (e.g. extreme rates of spread or energy release and novel wildfire phenomena), impacts (smoke emissions and their health impacts, carbon emissions, loss of lives, damage to ecosystem and infrastructure) or feedbacks (immediate fire–atmospheric-fire behaviour or carbon emission–atmospheric warming). The research questions outlined below were perceived as helpful to improve our understanding and ability to predict and manage wildfires and the resulting changes in ecosystems that we face.

##### Are existing fire behaviour and fire danger rating systems fit for purpose?

Most of our wildfire prediction systems, such as fire danger rating systems and models that estimate fire behaviour, are underlain by data gathered over 50 years ago. The most widely used to estimate surface fire spread is Rothermel’s quasi-empirical model of 1972 [30]. This model underlies the USA National Fire Danger Rating System (NFDRS) as well as those of Portugal, Greece, France, New Zealand and Italy [31]. It also serves as part of the *BehavePlus* fire behaviour prediction model [32], utilized globally by practitioners and researchers as well as other USDA-led models such as *FlamMap* and *Farsite* [33]. Similarly, developed well over half a century ago and still used operationally (e.g. USA NFDRS) and in research (e.g. [34]) is Byram’s 1959 equation for fireline intensity [35]. These equations have served extraordinarily well over decades and continue to serve well in many cases, as indicated by testing of the NFDRS against observations of fire occurrence and final fire size in the USA [36]. However, in a new world of increasingly extreme wildfire behaviour, driven by both extreme meteorological conditions (e.g. prolonged warming and drying) [34] and shifts in land management [37], workshop participants highlighted that the underlying data and equations in well-established models need re-testing for existing ecosystems that exhibit new extremes. It was noted that we need to test these for fuels in regions that have in the past been considered as less wildfire prone but are now experiencing increased wildfire and different wildfire behaviour (e.g. northern and central European conifer forests and the sub-Antarctic forests of South America). To achieve this, new fundamental data needs to be gathered that underpins new understanding.

Furthermore, attendees noted that if we are to advance our understanding and predictive capabilities in fire behaviour, we need to develop and undertake cross-scale investigations. Much of what we know about fire behaviour comes from different scales of experiments, from lab-based flammability and energy release studies through to field-scale measurements of fire behaviour. However, we are still largely unable to translate high-resolution, accurate small-scale measurements to larger-scale behaviour, especially due to the differences in fuel heterogeneity at different scales. This becomes significant in translating combustion physics and chemistry phenomena (i.e. processes) into real predictive tools that enable landscape-scale predictions in different fuel types. Participants recommended that cross-scale investigations of phenomena should be designed and undertaken by collaborating researchers because too often teams focus on one or the other scale, perhaps based on funding and disciplinary limitations, which fails to create results that are communicable across scales. Support of this research directive could translate to a transformational shift in global wildfire prediction and mitigation capabilities.

Along a similar theme, smouldering fire phenomena were identified as becoming ever more critical to understand, both because they have been entirely neglected in models that predict landscape fire behaviour and because more land underlain by organic soils and peat is under threat from wildfires (e.g. Las Tablas de Daimiel, National Park, Spain, 2009; Western Greenland, 2017; Saddleworth Moor, UK, 2018). It was highlighted that we need to build a solid understanding of smouldering fire behaviour in a range of ecosystem/fuel types because the transition from smouldering to flaming is not well understood and has never been included in models that predict initiation and behaviour of fires [38]. This is despite recognition that smouldering fires can hide deep underground for days and even a whole season before emerging back to the surface and initiating new wildfires [39].

A need for the development of a universal fire behaviour modelling system that would be fit for purpose across the globe was also noted. Such a universal fire behaviour model would not only enable those in land and fire operations to make the best estimates for fire management and mitigation but also open up huge potential for modelling intercomparison of current and future fire behaviour across the globe. Improved representation of human–fire interactions within such models was seen as needed to be able to inform fire-related policy [27,28]. Moreover, it was highlighted that such a model should seek to allow land management strategies to be included in modelling outputs. Examples given included development of fuel management strategies, prediction of prescribed fire behaviour, and enabling proposed rewilding and afforestation schemes to also predict potential fire behaviour before final land-use changes are approved.

Linked to fire behaviour are fire effects because different fire behaviour can have different short- and long-term effects on ecosystem and plant communities. Some fire behaviour models also predict tree mortality (e.g. *BehavePlus* and *FOFEM*, both USDA). These include observations and characteristics for some tree types (traits such as bark thickness and crown damage), which participants suggested should be expanded to consider global ecosystems, a wider range of species and functional types, and predictive capabilities for novel and extreme wildfire behaviour. Moreover, many plants have adaptations that support their survival following fire [40]. Participants noted that it is essential to explore whether these traits remain fit to respond to novel or more extreme fire regimes. Hence, not only do existing mortality prediction frameworks need both expansion to a much wider diversity of plants and testing under new fire regimes, but building capacity to define a greater range of fire adaptations within modelling systems was identified as being valuable for understanding long-term fire effects on our ecosystems.

##### How do fires behave in shrubland ecosystems?

Shrub vegetation is one of the most flammable fuel types across the world’s climate zones. The distribution of key heath and shrubland species (e.g. *Calluna vulgaris*, *Erica* sp., *Ulex europaeus*) is extensive, and in many of these cases, they are highly successful invasive species beyond their native western European distribution. Shrub-fuelled fires burn not only in shrub-dominated ecosystems such as northern temperate heathlands, moorlands, USA chaparral and South African fynbos but also often provide the surface fuels in forests and can carry rapidly spreading intense fires. Despite their global importance in fire regimes and increasing role especially in northern temperate regions, participants agreed that shrub fires remain the least well understood because their flammability is complex, often driven by live (instead of dead) fuels with high volatile contents [41–43].

Shrublands tend to regenerate quickly post-fire and can lead to a shift from less-resilient tree-dominated ecosystems to resilient shrub communities in areas that suffer recurrent fires. They are therefore capable of creating a positive fire feedback and leading to the expansion of wildfire-prone areas [44]. Increases in shrubland area and fuel load often occur due to land-use changes, particularly land abandonment (e.g. Iberian peninsula) [45]. Additionally, the issue of fuel type and load changes, particularly in respect to non-native invasive species, has been brought to the forefront of wildfire challenges following the 2023 Lahaina wildfires in Hawaii (fire spread through large areas of non-native grasses). They saw an enormous loss of human life and 2700 structures damaged and were discussed extensively during the workshops. Indeed, both the Lahaina wildfires and the UK wildfires in the summer of 2022 (shrub and grass ecosystems) had an unprecedented loss of property (for their regions) but were both small-sized wildfires in comparable fuel types. We discussed in the workshops how it is not only extreme climate-driven wildfire behaviour that leads to large losses of lives and infrastructure but also the changing nature of fuel types present (including their flammability and associated wildfire behaviour predictability). The need for a better understanding of the variability in shrub behaviour and spread was highlighted as critical by participants.

Participants agreed that at present, fire behaviour in shrubland and grass–shrub ecosystems, including representation of non-native species in different ecosystems and climates, is under researched and we do not have strong predictive capabilities. For example, current operational fire behaviour models designed in the USA are recognized to not adequately model fire spread even in USA chaparral fuels [46]. Challenges highlighted include capturing the spatial heterogeneity in shrub fuel biomass [47] and moisture, recently shown to be highly variable across the landscape [48]. Indeed, it has been suggested that fireline intensity varies greatly within relatively small plots, exhibiting areas of very low through to high intensity within an order of magnitude difference within a few metres and seconds [34]. This suggests that existing equations in models that predict fireline intensity need to include nonlinear variabilities to describe wildfire behaviour in shrublands. Another issue highlighted is that shrub-dominated ecosystems typically host plants that are highly volatile-rich, believed to contribute significantly to initiating fires and supporting high fire intensity and rapid spread. For example, terpene content has been considered a biochemical trait that enhances fire because it determines the amount of energy within the fuel. Therefore, the baseline chemistry of plants is also a fundamental driver of the combustion reaction [49]. This influences the energy content assigned to fuel models that sit within operational fire behaviour modelling systems. Recent research suggests that many shrub fuels actually have heat of combustion values that are above the limits that such systems will accept as input values [50]. Hence, even where fuel models are being improved based on new fundamental observations, these cannot be included within existing frameworks. As discussed, this highlights that existing operational systems have not been built with shrub fuels in mind nor have they been underlain by fundamental measurements of shrub fuels or the fire behaviour they support.

##### How can we better predict how fires spread through the WUI?

The WUI has been the fastest-growing land-use type in the USA, experiencing a 41% increase in the number of new houses from 1990 to 2010 [51]. Global change, including climate, land use, and population and WUI growth, will be critical in shaping the wildfire threat at these interfaces [26,52]. There was a consensus across participants that modelling fire spread through the WUI, not just to the edge of the interface, remains an elusive challenge [53,54]. We urgently need to understand fire behaviour within the WUI, particularly where communities border complex, continuous vegetation arrangements that can act as fuel for wildfire spread.

It is thought at least 50% of ignitions in the WUI are caused by firebrands [55]. Firebrand transport has been well researched, but the mechanisms by which firebrand exposure leads to ignition of recipient fuels were perceived as largely unknown [56]. Not only can we not effectively model firebrands, but we also cannot observe or map firebrands via UAV or high-resolution satellite imagery, further limiting our mechanistic understanding of firebrands and their representation in fire spread models [57,58]. The ignition probability of recipient fuels and their transition from smouldering to flaming combustion was highlighted as a complex process that needs to be understood to model firebrand ignition in the WUI. Existing fire spread models are largely semi- or fully empirical models of steady flame spread [59]. We cannot currently predict how a fire will respond to the likes of fire breaks or WUI structures like wooden fencing [56]. As such, participants stated that it is also difficult to quantitatively assess and predict the effectiveness of fuel treatments. Research is also needed to assess over what scale fuel treatments are needed to reduce risk in WUI/RUI areas. Although there is some evidence that fuel treatments have reduced the ingress of wildfires into urban areas [60], the fuel treatment application area, treatment type and treatment frequency have received little attention.

### (ii) Increasing human and ecosystem resilience to fire

After a long period of fire exclusion in western societies, it is now well recognized that fire is a critical component of many ecosystems, and there is a move towards thinking about how we can ‘coexist with fire’ [61]. Fuel management, adaptation and mitigation are all integral to increasing fire resilience: accepting that fire is part of the landscape while reducing the risk of extreme wildfires. To do this, we need to understand the role of fire within the Earth system: how fire behaviour and impacts can compound and cascade with other hazards and the capability of ecosystems to adapt to changing fire behaviour.

Increasing fire resilience is as much a social and political issue as it is a scientific one, and there are many opportunities for transdisciplinary collaborations. The wildland–urban interface is the nexus of human–fire interactions and is a key area for research (e.g. risk communication and risk reduction strategies). The following research questions were identified by participants as key research gaps toward increasing human and ecosystem resilience to fire.

#### How can we minimize structural damage from wildfires?

Devastating loss of homes in recent years were seen to provide the impetus for focusing research efforts on minimizing structural damage from wildfires in the WUI/RUI. Participants highlighted a need for fundamental science to understand how structural damage occurs through direct flame contact (e.g. structure-to-structure ignition) and firebrand ignition in order to develop prevention strategies [62–64]. To achieve this, it was agreed that we need to connect existing observational data and experimental data to develop a joint evidence base of preparation strategy effectiveness across the continuum from process-based understanding to validation of models using real fire data. Collation of such existing knowledge gaps on the impacts of wildfire exposure, such as how the layout of buildings within the WUI can impact structural damage, was identified as essential to form priority research questions [65,66]. It was acknowledged that in general, less is known about informal settlements in this respect [67] and should be a targeted area of research.

#### What are the hydrological consequences of fire, such as erosion events triggered by rainfall or water quality impacts, and how can we adapt/mitigate?

Post-fire hydrological impacts were seen as a complex but critical global challenge. Extreme rainfall and erosion following wildfire events can trigger debris flows and short- and long-term water quality impacts. Water quality impacts can manifest through runoff of ash, sediment and toxic metals into surface waters [68–70] and damage to infrastructure networks, such as water treatment and distribution systems [71].

Many countries experience post-fire debris flows and erosion, and some such as the USA have developed specific models and strategies to reduce the risk of these impacts [72]. Much of the research on extreme post-fire erosion by water (debris flows), however, is based on geologically young, susceptible terrain such as the western USA [73] and much of the research on post-fire water contamination is based in forested terrain [74]. It was commented that it remains unclear to what degree findings can be translated to other terrain types/land covers that see increasing fire exposure [69,75].

It was also noted that in general, the long-term hydrological consequences of wildfire are much less known compared to the immediate post-event impacts [72]. Furthermore, modelling of post-fire impacts was identified as a complex challenge due to the coupled nature of catchment-scale processes across spatiotemporal scales and difficulties representing post-fire processes, like ash mobilization [72,76]. Despite the WUI being the location where people may be most affected by contaminated drinking water, it was noted that very little is known about the addition of WUI contaminants from burning of infrastructure. Participants suggested that post-fire hydrological consequences should be assessed through a multi-hazard lens to assess infrastructure thresholds, forecast multi-hazard events (including cascade and compound hazards) and develop adaptation and resilience strategies [77].

#### How should prescribed fires be used in different ecosystems to maintain ecosystem health and reduce risk of severe wildfires?

Fire is a natural part of many ecosystems, and humans have long been using fire to tend the land [22,78]. Traditional uses of fire across many different countries are carried out in different ecosystems and for different reasons, from usage by Indigenous people over millennia tending to the land through to use in the management of northern temperate heathlands over recent centuries in places like Norway and the UK [79,80]. In countries such as the USA, Canada, Australia and Spain, prescribed burning has been carried out specifically to reduce fuel loads and for ecological benefits, such as increasing species richness and biodiversity [81].

The impact of prescribed fire can vary significantly depending on the frequency, intensity and suitability of its use in specific environments. Prescriptions of fire (where, when and how burns should take place to achieve their objectives) for different ecosystems were highlighted as being important to identify and reduce the likelihood of negative impacts [82]. The need for fuel reduction strategies to reduce wildfire risk is an important global theme; however, climate change is also reducing the annual window for safe use of prescribed fire [83]. Smoke and air quality concerns are also significant factors limiting prescribed burning opportunities on otherwise suitable days [84].

Participants agreed that it remains difficult to evaluate the potential positive and negative effects of prescribed burning (compared with other land management strategies and wildfire) due to the longitudinal nature of post-fire impacts that typically exceed research funding cycle lengths [85]. There was consensus regarding the urgent need to understand how effective and environmentally and socioeconomically sustainable the use of prescribed burning is compared to other land management approaches in wildfire risk reduction and ecosystem management. Furthermore, the need for specific prescribed fire research to address knowledge gaps where wildfire-based science and applications (e.g. fire behaviour models) have not proven appropriate was highlighted [86].

### (iii) Understanding the atmospheric and climate impacts of fire

Atmospheric and climate responses to wildfires can be considered from a local to global perspective, where smoke and emissions know no boundaries. One of the perceived greatest global challenges in wildfire is understanding and mitigating the health impacts from wildfire smoke. The 2023 northern hemisphere wildfire season was discussed as demonstrating how smoke can have far-reaching impacts thousands of kilometres away, as was seen when the USA eastern seaboard was impacted by the wildfires in Quebec. Indonesia was regarded as a vital case study of how peatland fires can cause a deterioration of air quality and impact health not only nationwide but beyond [87], as seen by the return of the transboundary haze across Southeast Asia. Globally, many countries are vulnerable to air quality deterioration by regional and continental smoke dispersion, and it was noted that there is currently no international framework to mitigate these impacts. Another major challenge identified was quantifying wildfire emissions, because intertwined processes and their feedback make it difficult to estimate the overall consequences of fire on our changing climate. Research questions outlined by the participants below seek to address significant knowledge gaps using different methodological approaches that help tackle these key challenges.

#### What are the emissions from high-latitude/peatland/smouldering fires and how might they change?

Peatlands store approximately 25% of the world’s soil carbon and are most prevalent in the northern high latitudes [88]. High-latitude peatlands can be a large source of methane emissions [89], emitting *ca* 36 Tg CH_4_-C per year [90], while disturbed or degraded peatlands can also be a significant CO_2_ source [91]. Disturbances such as smouldering fires in organic soils release CO_2_ into the atmosphere and affect the carbon balance of these ecosystems with global implications [92]. It was highlighted that it is essential to understand both present and future peatland emissions. However, the complexity of peatland–fire dynamics makes quantifying emissions challenging [93].

Advanced satellite technology for monitoring global wildfire occurrence and emissions now exists (e.g. NASA Fire Information for Resource Management System, European Commission Global Wildfire Information Systems and Copernicus Atmosphere Monitoring Service Global Fire Assimilation System). These help to understand the regional and global implications of biomass burning on daily emissions such as CO₂, CH₄ and N₂O [94] and help to reduce uncertainty, especially for northern forests and peatlands where combinations of remotely sensed, experimental and process-based modelling approaches are needed. Participants agreed that there must be a future focus in quantifying emissions due to the significant climate change impacts in these regions [5].

Despite their critical role as drivers of climate change, peatland and smouldering fires were noted as being not yet included effectively in Earth system models and therefore climate projections [95,96]. Collaborative efforts towards model development were seen as required whether we are to forecast emissions, smoke transport and carbon feedbacks [7,92]. Focused areas identified were understanding the role of permafrost thaw [7], evaluation of empirical models [97], the role of peat bulk density [98], understanding of burn severity in relation to burn depth [92] and smouldering fire phenomena [38] in relation to peatland–fire dynamics.

#### What are the impacts of smoke and emissions on human health and how can they be managed?

Wildfire smoke exposure has detrimental impacts for human health, estimated to contribute to over 340 000 premature deaths each year globally [14]. Smoke and emissions can affect people through immediate short-term exposure during a wildfire and long-term from significant or repeated exposure events [99]. There is an identified current lack of knowledge on the impacts on human health, particularly by repeated exposures across fire seasons, for people living near wildfires or prescribed fires as well as for firefighters [100–102]. The consensus is that wildfire smoke can be more harmful than smoke from prescribed fires due to larger spatiotemporal extent and potential range of pollutants [103,104], yet participants noted more research is needed to evaluate the impact of repeat exposure to smoke on health and the health trade-offs between prescribed and wildfire smoke [105]. Knowledge gaps included the toxicology of different fuel sources, in particular from WUI fuels, i.e. burning of structures within the WUI [101,106]. It was noted that there is also very little documentation of mental health impacts from wildfire smoke events [102] and understanding perceptions on smoke as a hazard has received little attention but is particularly relevant given the increasing frequency of exposure [107]. The effectiveness of prevention and mitigation strategies for reducing smoke impacts was perceived to require validation [101].

The global reach of smoke impacts has received a lot of attention in recent wildfire seasons, but research on smoke governance and international frameworks for mitigation of smoke’s effects is needed to address this challenge. For example, the smoke over the northeastern USA in 2023 originated from wildfires in Quebec nearly 2000 km away and is evidence that large populations not typically exposed to wildfire can still be negatively impacted [108]. Smoke transport is spatiotemporally variable based on fuel type, amount and behaviour of the wildfire [109]. Participants agreed that researchers that specialize in atmospheric transport and dispersion modelling must come together with those with Earth observation modelling capability at a global scale as this could provide opportunities in tracking smoke exposure across spatiotemporal scales using remote and ground-truth data [105]. Likewise, additional inputs from experimental and observation-based research were noted as required to improve such models. Moving forward, there is a clear need for real-time coupled smoke transport model development to mitigate and provide clear advisory alerts in respect to human health.

#### What is the overall net climate forcing of shifting fire across the globe?

Climate change impacts wildfires in numerous ways (e.g. increasing lightning-caused fires [110], altering vegetation [111] and flammability [52]). In turn, different fire behaviours and frequencies can alter carbon fluxes as well as other greenhouse gases over relatively short timescales. Approximately 8 billion tonnes of CO_2_ are estimated to be emitted to the atmosphere annually from global fires [3]. Wildfires can also cause indirect changes to the climate, for instance, the impact that wildfires have on species composition and vegetation shifts, which influences the climate through productivity, carbon storage and fire resilience [3,112]. Wildfires can therefore act to accelerate or diminish changes to the climate through fire–climate feedbacks [3].

Other areas for fire–climate feedback suggested throughout the literature include changes in physio-chemical and biological soil properties, ecosystem properties, transpiration rates, trace gases and aerosols, deposition and sequestration of pyrogenic carbon, altering atmospheric composition and chemistry and changing surface albedo [3,110,112,113]. As global fire regimes shift in response to the changing climate, the challenging key question arises: What does this mean for emissions, carbon fluxes and their net climate forcing?

Participants noted that of particular interest is changing wildfire forcings in the Arctic and high-latitude regions where climate amplification results in changes occurring at rates faster than anywhere else in the world [29]. This is already reflected in enhanced fire activity in these areas [3]. As fires are expected to become increasingly common at high latitudes [114], there will likely be many important feedback at play, pivotal to our understanding of climate–fire forcing. For instance, as fires consume vegetation and affect the thaw depth of underlying permafrost, there is likely further loss of carbon storage and increase of GHG emissions that would further enhance warming in the area. Furthermore, smouldering fires may consume organic matter in high-latitude peatlands and release soil carbon. On the other end of the scale, wildfire-driven high-latitude changes in albedo may lead to negative climate–fire feedbacks and result in a net cooling effect over longer periods (>5 years), despite initial short-term positive feedbacks [115,116].

Although there is an increasing awareness of the importance of understanding climate–fire feedbacks, it was noted that studies are often limited to specific regions, and much remains largely unknown on a global scale with interactions between wildfire and climate being complex and occurring over multiple timescales [117]. We are currently limited by knowledge of process understanding and modelling capabilities. Despite the importance, simulations of global fire in Earth system models vary greatly based on what is implemented in the models, which were acknowledged as inevitably simplified and based on outdated assumptions. Additionally, many fire models remain unable to produce observations on a global scale [113,115]. Participants agreed that efforts are therefore needed to advance global fire models. This needs collective research using Earth observation approaches, improving climate modelling and global fire models. Collaborations including improved observational data and taking advantage of new satellite sensors could accelerate the changes required and push towards better understanding the net climate forcing of shifting global fire.

### (c) Wildfire challenges require more than science can solve alone

While this opinion piece presents wildfire natural science research needs through the lens of academic and government wildfire scientists, linkages with the social sciences, arts and humanities were flagged throughout all the workshops as being essential to move forward in many areas of wildfire research and governance. Particularly in respect to determining how science can inform management, policy and governance, and society. During the workshops, discussions were clear that connections with the social sciences and disciplines such as human geography are needed if we are to determine and evaluate ways to improve people’s understanding of fire and adapt to increasing wildfire risk, at the same time as improving risk communication and enhancing wildfire preparedness as examples. To this end, we include some of the thoughts from participants below, which we hope will allow greater nuanced thinking between disciplines (and beyond conventional research groups) so that we can provide the best insight of use of management, policy and governance, and society. The recent work by Copez-Gerbits *et al*. [25] offers valuable insights here on understanding and enabling transdisciplinary and transformative research. Researchers within academic and government institutions in particular must actively question long-held assumptions and address power imbalances to enable co-development of knowledge outside of rigid institutions and practice problem-centred research.

#### (i) Risk communication and reduction

When we discuss risk communication, it should be noted that it is inappropriate (and has proven unsuccessful to date) to develop research questions from an academic standpoint only. Local citizens, communities, land managers and associations must be co-developers to prevent the pitfall of creating expert-driven, top-down actions that continue to fail to reach their desired outcomes. Transformative change can come from moving beyond the wildfire risk paradigm to broader wildfire communication and coexisting with fire, thorough recommendations for which can be found in [25,118]. That said, risk communication was identified by both academic and government researchers as a critical area needing research and funding. This might include evaluating ongoing wildfire risk communication techniques and identifying best practice that could be reproduced or adapted to further contexts or specific ‘vulnerable groups’.

Reducing wildfire risk in the WUI was identified as being particularly challenging because the WUI is a socioecological system that requires both fundamental natural and social science and involves a diverse range of actors, including government policy (e.g. [119–121]). Moreover, social science research is only beginning to untangle the multitude of factors involved in the uptake and maintenance of risk reduction efforts and protective behaviours, and case studies have sometimes demonstrated opposite results [122]. Participants highlighted that recent wildfire seasons have demonstrated the need to develop targeted risk communication for preparedness under unforeseen circumstances, such as when there is no time for official warnings or when things go wrong [123,124]. This can only be achieved via co-creation of systems informed by academics in the sciences and humanities, together with government think tanks, management agencies and associated decision-makers.

There is also a need to move beyond solely thinking about risk communication. Participants identified more research and funding is needed to support understanding of effective wildfire risk reduction practices. From a social science perspective, risk reduction is most effective when it is ‘bottom-up’ and ‘community-based’ as it can help strengthen local capacities, increase ownership of risk reduction efforts and enhance community resilience [125]. Participants agreed that efforts are therefore needed to embed, evaluate and develop wildfire risk reduction practices that are locally led, inclusive and sustainable.

#### (ii) People’s understanding of fire

In this digital age, people have access to more information than we have ever had, including real-time information on unfolding wildfire disasters across the world. There is a need to understand how people’s understanding of fire has evolved alongside developments in social media [126], for example, how well can social media be used to communicate effectively, and to what extent can it lead to misconceptions? [127].

Many historically wildfire-prone countries such as the USA and Spain have undergone a long process of evolving risk communication strategies, from aggressive suppression to slowly increasing acceptance of the role of fire in the landscape [128]. People’s understanding of wildfire risk in mid-latitude temperate and other historically less fire-prone countries lags behind. Participants highlighted that both historical and emerging wildfire-prone regions alike often share histories of wildfire suppression that cannot be sustained under climate change and land cover change, and there are opportunities for international collaboration to address this priority of adapting to coexisting with fire [126,129,130].

## 4. Conclusion

Over recent years, there have been a range of publications suggesting a paradigm shift is required in respect to our understanding of wildfires [22,25]. We have summarized here the shared research priorities as perceived by academic and government wildfire scientists from a historically wildfire-prone (USA) and an emerging wildfire-prone (UK) country during a series of workshops. Such events offer important opportunities to bring people together to work towards addressing these challenges. The identified priority areas aimed to address critical remaining and emerging research gaps for the changing wildfire regimes across many parts of the globe driven by climate change and other anthropogenic impacts. Our findings provide a perspective to feed into a transdisciplinary outline of priorities in wildfire research across the diversity of disciplines, sectors, countries and cultures that will be needed to address future wildfire challenges under changing fire regimes.

Workshop participants identified fire behaviour and danger, WUI/RUI, fire ecology and severity, and smoke and emissions as key themes, of which fire behaviour was seen as the central connecting theme allowing critical advances to be made across all areas. In terms of *predicting fire occurrence, fire behaviour and fire impacts*, priorities identified were updating fire behaviour and fire danger systems to cover new extremes, fire spread in the WUI, and fuels and regions that are now experiencing increased fire and novel fire behaviour. This requires both gathering new fundamental data and developing new understanding. To *increase human and ecosystem resilience to fire,* the connected underlying social, political, economic, engineering and natural environmental factors need to be addressed together. Here, transdisciplinary collaborations are particularly important, to minimize, for example, the rising structural damage from fire, impacts on silvicultural and agricultural resources or ecosystem services such as water provision. We also urgently need to understand how environmentally and socioeconomically sustainable prescribed burning can be used into the future for wildfire risk reduction and ecosystem management. Finally, *understanding the atmospheric and climate impacts of fire* was seen as a priority, particularly for predicting and mitigating the health impacts from wildfire smoke. We require a more complete understanding of the processes and feedback that determine the overall consequences of fire on our changing climate both locally and globally. For example, the effects and trade-offs between smoke from wildfires and fuel reduction burns under changing environmental and socioeconomic conditions need attention. Advances in satellite observations, computing power, artificial intelligence and Earth system models may together lead to a better understanding of the net climate forcing of changing global fire regimes.

## Data Availability

This article has no additional data.
